# Supplementary material for: A Narrative Review on the Approach to Antimicrobial Use in Ventilated Patients with Multidrug Resistant Organisms in Respiratory Samples—To Treat or Not to Treat? That Is the Question
Source: Antibiotics (Basel). 2022 Mar 27;11(4):452. doi: 10.3390/antibiotics11040452 (PMC9031060; doi:10.3390/antibiotics11040452)
Supplement: Supplementary file 1 [file antibiotics-11-00452-s001.zip › antibiotics-1652937-supplementary.pdf]

Literature Search Strategy

Database: Ovid MEDLINE(R) <1946 to March 14, 2022>
Search Strategy:
----
1 Drug Resistance/ or multidrug resistant.mp. (82522)
2 Drug Resistance, Multiple, Bacterial/ or MDRO.mp. (24445)
3 multidrug-resistant.mp. (35199)
4 Drug Resistance, Microbial/ or Antimicrobial Resistance.mp. (80283)
5 multidrug-resistant.m_titl. (9896)
6 multidrug resistant.m_titl. (9896)
7 antimicrobial resistance.m_titl. (7143)
8 MDRO.m_titl. (26)
9 Pneumonia, Ventilator-Associated/ or VAP.mp. (6702)
10 VAP.m_titl. (313)
11 ventilator associated pneumonia.mp. (5132)
12 ventilator associated pneumonia.m_titl. (2540)
13 decision.mp. or Clinical Decision Rules/ or Clinical Decision-Making/ or Decision Making/ (356440)
14 treatment strategy.mp. (23659)
15 strategy.mp. (492952)
16 decision.m_titl. (48261)
17 strategy.m_titl. (56222)
18 interpretation.mp. (323180)
19 interpretation.m_titl. (24540)
20 1 or 2 or 3 or 4 or 5 or 6 or 7 or 8 (169130)
21 9 or 10 or 11 or 12 (8138)
22 13 or 14 or 15 or 16 or 17 or 18 or 19 (1135838)
23 20 and 21 and 22 (68)
****

88 articles found

PUBMED 1951 to March 14, 2022

((drug resistance) OR (multidrug resistant) OR (MDRO) OR (multidrug resistant organism) OR (multidrug-resistant) OR (antimicrobial resistance)) AND ((VAP) OR (ventilator-associated pneumonia) OR (ventilator associated pneumonia) OR (ventilator associated tracheobronchitis)) AND ((decision) OR (decision making) OR (clinical decision rules) OR (treatment strategy) OR (strategy) OR (interpretation))

330 articles found
